# Next Generation Sequencing Technology in Lung Cancer Diagnosis

**DOI:** 10.3390/biology10090864

**Published:** 2021-09-03

**Authors:** Calin Cainap, Ovidiu Balacescu, Simona Sorana Cainap, Laura-Ancuta Pop

**Affiliations:** 1 Department of Oncology, “Iuliu Hatieganu” University of Medicine and Pharmacy, 400012 Cluj-Napoca, Romania; Calin.Cainap@umfcluj.ro; 2 Ion Chiricuta Institute of Oncology, 400015 Cluj-Napoca, Romania; 3 Department of Genetics, Genomics and Experimental Pathology, The Oncology Institute Ion Chiricuta, 400015 Cluj-Napoca, Romania; ovidiubalacescu@iocn.ro; 4 Pediatric Clinic no 2, Department of Pediatric Cardiology, Emergency County Hospital for Children, 400177 Cluj-Napoca, Romania; 5 Department of Mother and Child, “Iuliu Hatieganu” University of Medicine and Pharmacy, 400013 Cluj-Napoca, Romania; 6 Research Center for Functional Genomics, Biomedicine and Translational Medicine, “Iuliu Hatieganu” University of Medicine and Pharmacy, 400337 Cluj-Napoca, Romania; laura.pop@umfcluj.ro

**Keywords:** lung cancer, next generation sequencing, diagnosis

## Abstract

**Simple Summary:**

Lung cancer is still one of the most commonly diagnosed and deadliest cancers in the world. Its diagnosis at an early stage is highly necessary and will improve the standard of care of this disease. The aim of this article is to review the importance and applications of next generation sequencing in lung cancer diagnosis. As observed in many studies, next generation sequencing has been proven as a very helpful tool in the early detection of different types of cancers, including lung cancer, and has been used in the clinic, mainly due to its many advantages, such as low cost, speed, efficacy, low quantity usage of biological samples, and diversity.

**Abstract:**

Lung cancer is still one of the most commonly diagnosed cancers, and one of the deadliest. The high death rate is mainly due to the late stage of diagnosis and low response rate to therapy. Previous and ongoing research studies have tried to discover new reliable and useful cbiomarkers for the diagnosis and prognosis of lung cancer. Next generation sequencing has become an essential tool in cancer diagnosis, prognosis, and evaluation of the treatment response. This article aims to review the leading research and clinical applications in lung cancer diagnosis using next generation sequencing. In this scope, we identified the most relevant articles that present the successful use of next generation sequencing in identifying biomarkers for early diagnosis correlated to lung cancer diagnosis and treatment. This technique can be used to evaluate a high number of biomarkers in a short period of time and from small biological samples, which makes NGS the preferred technique to develop clinical tests for personalized medicine using liquid biopsy, the new trend in oncology.

## 1. Lung Cancer

Lung cancer remains one of the most common cancers diagnosed in 2020, and one of the deadliest cancer types. In Europe, the lung cancer incidence rate is 97.6 (men) and 38.3 (women), with a mortality rate of 81.7 (men) and 29 (women), respectively [1]. In Romania, the incidence is lower in women (28.5) and higher in men (105.3) than the European incidence rate. The same trend is also observed in mortality, 24.8 (women) and 95.6 in men [1]. An increased number of deaths due to lung cancer is mainly due to late-stage diagnosis, mostly because this cancer shows no symptoms in its early stages. 

There are several risk factors associated with lung cancer, such as smoking, air pollution, radon exposure, occupational exposure to different chemicals, heredity susceptibility, radiation and diet [2]. Considering these risk factors, it has been observed that specific subtypes are correlated to exposure to specific risk factors. These subtypes are small cell lung cancer and non-small cell lung cancer. Small cell lung cancer (SCLC) represents about 15% of lung cancers and is correlated mainly to smoking. Non-small cell lung cancer (NSCLC) has three main subtypes and accounts for 85% of lung cancer diagnoses. These subtypes are adenocarcinoma, squamous cell carcinoma and large cell carcinoma [3].

Lung cancer is diagnosed at the late stage mainly because in the early stage it presents no symptoms, and patients approach a doctor only when they experience chest pain, persistent cough, and weight loss. Due to this fact, it is very important to identify reliable methods for screening patients with a high risk for lung cancer. In 2018, the National Comprehensive Cancer Network (NCCN) defined low-dose computer tomography (LDCT) as an early screening method for high-risk lung cancer patients [4]. In a German trial, the LDCT screening helped reduce mortality in women with lung cancer [5]. Two main disadvantages of this technique are that it is recommended for a specific range of patients, mainly people that are smoking or are between 50 and 80 years old, and that there are difficulties in evaluating the correct size and number of lung nodules on CT scans, and sometimes these nodules are benign. To overcome these limitations, new methods for lung screening and diagnosis have been developed [6,7]. It is possible to improve lung cancer screening and diagnosis by using LDCT in combination with different biomarkers, either from serum or blood [8]. On the other hand, due to a lower treatment success rate for late-stage lung cancer, its mortality is relatively high. Moreover, lung cancer patients who smoke present a higher number of somatic mutations, which can give rise to a higher number of cancer-driven mutations [9]. Computer tomography is still the main method used for lung cancer screening and diagnosis [10], and the application of genetic testing is mainly used for treatment selection and guidance. Studies have shown that genetic testing using NGS has helped identify at least one actionable target that could be used for targeted therapy [11,12,13]. Additionally, it was observed that patients treated with targeted therapies show better survival and response rates [14]. For lung cancer patients, mainly NSCLC, treatment is still based on chemotherapy for initial stages, but in local, advanced or metastatic disease, biomarker testing for different genes (EGFR, ALK, KRAS, ROS1, BRAF, NTRK1/2/3, MET, RET and PD-L1) helps patients benefit from specifically targeted therapies (anti-EGFR, anti-ALK or anti-ROS) and immune checkpoint inhibitor therapy [15].

Therefore, by using and corroborating the data provided by next generation sequencing (NGS) assessment, the early diagnosis and guidance of treatment for lung cancer have become more precise.

## 2. Next Generation Sequencing

Next generation sequencing (NGS) is a comprehensive technology used for sequence (DNA) and gene expression (RNA species) analysis [16,17,18]. The NGS technique was developed to overcome the Sanger sequencing limitation, but it evolved into being used in all areas of genomic research, starting with DNA, RNA, miRNA, ChIP and methylation sequencing [19,20,21]. As with any technique, NGS has multiple advantages that have made it an essential tool in all areas of research and in the clinic [22]. However, even after over 15 years of development, this technique has some disadvantages, such as the need for powerful bioinformatics tools and specialized personnel for both experimental and data analysis [20]. Some advantages and disadvantages of NGS are presented in Table 1.

The data provided by NGS have proven valuable and reliable for both research and in the clinic to improve the diagnosis, prognosis, and treatment of several diseases [23,24,25,26], and are widely used in the oncology field [27,28]. In lung cancer, this technique has been used for early diagnosis biomarker identification, targeted treatment decisions, and identification of causative mutations [29,30,31,32,33,34].

## 3. NGS in Lung Cancer Diagnosis

Lung cancer diagnosis is challenging in the early stages because patients do not present any symptoms, or symptoms are shared with other pulmonary diseases. In addition, classic techniques for lung cancer diagnosis have many false-negative results due to different reasons, such as quality and quantity of the samples or sensitivity of the test [35]. Here, NGS can be beneficial due to its high sensitivity and specificity, using low amounts of sample. Additionally, NGS can determine an increased number of alterations simultaneously from the same quantity of sample. Therefore, NGS has been applied with success in the identification of lung cancer-specific mutations in paraffin-embedded tissue samples, with a higher rate than standard PCR testing [36,37]. Recently, studies have shown that NGS can effectively be used to identify specific lung cancer mutations in circulating tumor DNA, in a liquid biopsy sample [38,39,40,41]. The main applications of NGS in the clinic are related to genomics, transcriptomics and epigenomics. When using whole genome, whole exome or targeted DNA analysis, specific information on point mutations, copy number alterations, small indels or structural variance alterations can be identified. RNA seq analysis can provide information related to gene fusions, alternative splicing, differential expression or RNA editing, while Bisuphite seq or ChIP seq are used for the identification of the methylation profile, histone modification or transcription factor binding alterations. 

These important advantages demonstrated by NGS in the evaluation of the alterations related to lung cancer diagnosis have created a new opportunity for the development of commercial kits and assays specific to lung cancer. One such kit is the NextDaySeq-Lung panel, developed by Beijing ACCB Biotech (Beijing, China), with primers for the amplification of EGFR exon 18, 19, 20, 21, KRAS exon 2, 3, PIK3CA exon 9, 20, and BRAF exon 11, 15. The mutations in the KRAS gene can predict the efficiency of EGFR-tyrosine kinase inhibitors [42]. Recently, it was observed that most patients that developed resistance to TKIs have different EGFR mutations [43,44]. Mutations in BRAF can be correlated to response to BRAF/MEK inhibitors in NSCLC patients [45,46,47], while PIK3CA mutations could render SCLC patients sensible to triciribine treatment [48]. In addition, there is a study that uses alpelisib, a PIK3CA inhibitor, for breast cancer PIK3CA mutated patients, who have shown better survival than that of other treatment [49], which could be implemented in lung cancer as well. The NextDaySeq-Lung panel has been used in several studies and has demonstrated better results than Sanger sequencing or qRT-PCR [50,51]. Other gene panels specific for lung cancer focus on fusion alterations, based on RNA sequencing, and evaluate translocations, chromosomal inversions or interstitial deletions. One such panel is the Ion Ampliseq RNA fusion lung cancer panel offered by ThermoFisher Scientific, Waltham, USA, which targets 70 known fusion transcripts of ALK, RET, ROS1, and NTRK. This panel has shown high sensitivity and good concordance with the typical methods used for fusion testing [52]. For fusion testing, RNA seq has proven to be more sensitive and is used in parallel with DNA seq for mutation evaluation [53,54]. In addition, NGS has successfully been used to identify lung cancer patients that had MET exon14 skipping alterations [55]. Some other NGS lung cancer panels are presented in Table 2.

It is well known that cancer is considered a genetic disorder in which somatic mutations accumulate and give cancer cells the ability to over proliferate and avoid apoptosis [56,57]. Lung cancer is one of the cancers that exhibit a high degree of mutation burden and a high number of driven mutations [9]. Consequently, NGS is extremely useful, due to its many advantages, and the development of different NGS panels is implemented in the clinical setting. In lung cancer diagnosis, NGS is employed mainly in evaluating the gene alteration in key genes involved in the development of lung cancer. These genes are EGFR, BRAF, KRAS, HER2, ROS, ALK, PIK3CA, NTRK, RET and MET [58]. One example is using CGH NGS-based assay for assessing 51 FFPE samples of adenocarcinoma to evaluate its efficiency compared to standard mutation testing. The authors observed that 58% of wild-type patients presented alterations in one of these genes when using the NGS approach, making them suitable for targeted therapy [35]. In other studies, NGS was implemented for NSCLC diagnosis due to the small quantity of tissue samples, which is not suitable for traditional testing methods. Hagemman et al. successfully sequenced 209 samples of NSCLC using a 28 gene NGS panel and identified actionable mutations in 46% of the tested samples [36]. In the same line, Moskalev et al. used the 454 NGS system to evaluate EGFR and KRAS mutation in NSCLC samples with a low number of tumor cells. They were able to identify mutations with an allele frequency of 0.2–1.5%. When reevaluating 16 cases with low tumor cells that were wild type by Sanger, seven of them presented mutations in the EGFR gene at a frequency of 0.9–10% [37]. Another study compared an NGS panel, Sanger sequencing, and qRT-PCR in evaluating mutation in 138 NSCLC FFPE samples. The authors observed that NGS and qRT-PCR have a higher sensitivity than Sanger sequencing. NGS is better than qRT-PCR because it also provides information about the mutation sequence and allele frequency, and identifies mutations that are not in the hotspot area [50]. Liang et al. used a DNA methylation profile to develop a blood-based test for the early diagnosis of lung cancer. Their method presented a sensitivity of 75% for stage 1A and 85.7 for stage 1B lung cancer [59]. NGS has proven to be more sensitive and specific than FISH or IHC when analyzing fusion alterations in lung cancer, which are the main methods used for fusion detection. Lin et al. observed a positive rate of 92.7% for ALK rearrangement when using NGS, 82.4% for FISH and 94.5% for IHC, and a concordance of 87.3% of NGS results with IHC results. They also concluded that IHC fusion testing is better for screening, while NGS fusion testing is more accurate for predicting the clinical benefits of crizotinib treatment [60]. Another benefit of NGS is the fact that it also provides information on the exact fusion alteration, which is very important in evaluating the treatment and outcome of patients [61]. To overcome the problem of harvesting tissue samples from early-stage lung cancer sample patients, new challenges related to identifying novel non-invasive biomarkers are under investigation. One such example is the use of miRNA for the diagnosis of lung cancer. miRNA sequencing was used to identify specific miRNAs for adenocarcinoma and SCLC. Jin et al. were able to identify miR-181-5p, miR-30a-3p, miR-30e-3p and miR-361-5p as being specific for adenocarcinoma, and miR-10b-5p, miR-15b-5p and miR-320b for SCLC (small cell lung cancer) [62]. In addition, taking advantage of the many benefits of NGS, oncology researchers have developed liquid biopsy testing for lung cancer diagnosis [63]. Leighl et al. observed a very high concordance for NGS results from cfDNA and tissue DNA in untreated metastatic NSCLC [64]. The same was observed by Mack et al. when analyzing 8388 cases of NSCLC [65]. NGS testing was successfully recommended in lung cancer diagnosis by different expert panels [66] and oncology organizations [67]. Gray et al. performed a thorough survey of the relevant literature regarding liquid biopsy and observed that the advantages of NGS have helped to develop different assays using liquid biopsy samples for the early diagnosis, treatment selection, minimal disease detection, monitoring treatment efficacy and evaluation of tumor burden in lung cancer [63]. Sueoka-Aragane et al. observed that the analysis of ctDNA by NGS could be a promising tool for the evaluation of the efficacy of osimertinib in NSCLC with EGFR T790M mutation [68]. Table 3 presents studies correlated to the performance of NGS technology in the diagnosis and screening of lung cancer [69,70].

The research area using NGS for lung cancer diagnosis has been extensively developed and has made this technique valuable for different clinical trials on lung cancer. Data from clinicaltrial.gov include 98 trials on lung cancer that use NGS (https://clinicaltrials.gov/ct2/results?cond=Lung+Cancer&term=next+generation+sequencing&cntry&state=&city=&dist=, accessed on 25 July 2021). Some of these trials are already complete; others are recruiting or enrolling. There are clinical trials that evaluate the possibility of NGS to identify mutations in very small samples (NGS NCT02420405), or improve the personalized treatment (NCT02281214). Table 4 presents data on the clinical trials using NGS for lung cancer diagnosis. 

In addition, recent studies have shown that by implementing NGS-based testing, clinics can reduce the cost required for evaluating biomarkers specifically for targeted treatments or agnostic therapy implementation. NGS-based testing can reduce total testing cost by EUR 30–1249 depending on how comprehensive the analysis is, when compared to RT-PCR technology [80]. 

## 4. Conclusions

NGS has successfully been used both in research and in the clinic, and has become one of the main tools in lung cancer diagnosis, showing better results that standard techniques used for lung cancer diagnosis, and being able to identify lung cancer-specific alteration in a variety of biological samples such as blood, plasma, fresh frozen or FFPE tissue, urine or other bodily fluids, even where the nucleic acid content is limited and where classic methods fail. In addition, the cost of NGS is lower than that of standard testing methods, which makes this technique appealing for the implementation of different agnostic therapies, targeted therapies and immune checkpoint inhibitor therapies. Its success was demonstrated in different clinical studies that were developed to obtain better methods for lung cancer diagnosis. The NGS technique has become the primary tool for investigating different types of samples and different subtypes of lung cancer, being implemented in mutation evaluation and fusion alteration identification, due to its great advantage over FISH and IHC, which are techniques that can have inconsistent results due to the expertise of the pathologist, and cannot be used on other types of samples, except tissue. As with any technique, NGS still has its limitations, mostly related to the amount of data obtained, and the need for a big data storage capacity and a good bioinformatics team.

Nevertheless, the advantages of NGS make it ideal to be used for evaluating a high number of biomarkers in a short period of time, from small biological samples, and at a low price. Therefore, NGS should be the preferred technique to develop clinical tests for personalized medicine using liquid biopsy, the new trend in oncology.

## Figures and Tables

**Table 1:** Advantages and disadvantages of NGS technology.

Advantages	Disadvantages
Low price	Need for specialized software and computers for data analysis
Short time from library preparation to results	No standardization or availability of standardized material for clinical application
Variety of applications	Still expensive in some developing countries
Useful both in research and clinic	
High number of commercially available NGS platforms and specialized kits	

**Table 2:** Some commercially available NGS panels for lung cancer testing.

Name	Company	Type of Sequencing	Gene Targeted	Target Approach for Gene Fusion Analysis	Input Nucleic Acid (ng)	Type of Test
AccuFusion	Paragon Genomics, Hayward, USA	RNA fusion	ALK, CIT, EML4, FGFR1, MBIP, MET, NRG1, NTRK1, NTRK3, PDGFRA, RET, ROS1, TACC3.	Amplicon based	10	Diagnosis and treatment selection
OmniFusion	Paragon Genomics, Hayward, USA	RNA fusion	ALK, CIT, MBIP, MET, NRG1, NTRK1, NTRK3, PDGFRA, RET, ROS1, TACC3	Amplicon based	25	Diagnosis and treatment selection
Ion AmpliSeq™ RNA Fusion Lung Cancer Panel	ThermoFisher Scientific, Waltham, USA	RNA fusion	ALK, RET, ROS1, and NTRK	Amplicon based	10	Diagnosis and treatment selection
QuantideX^®^ NGS RNA Lung Cancer Kit	Asuragen, Austin, USA	RNA expression and fusion	ALK, ROS1, RET, FGFR3NTRK1, NTRK3, NRG1, FGFR1, FGFR2, MBIP, PDGFRA, MET, ABCB1, BRCA1, CD274, CDKN2A, CTLA4, ERCC1, ESR1, IFNGR, ISG15, MSLN, PDCD1, PDCD1LG2, PTEN, RRM1, TDP1, TERT, TLET3, TOP1, TUBB3, TYMS	Amplicon based	10	Treatment selection
TruSight RNA fusion panel	Illumina, San Diego, USA	RNA seq	507 fusion-associated genes	Hybrid capture based	10 total RNA20–100 FFPE RNA	Treatment selection
Archer fusion plex Comprehensive Thyroid and Lung	ArcherDX Inc, Illumina, San Diego, USA	RNA seq	gene fusions, SNV, indels, splicing and gene expression in 36 genes	AMP based	10 ng	Diagnosis
Archer fusion plex Lung kit	ArcherDX Inc, Illumina, San Diego, USA	DNA and RNA seq	EGFR vIII and MET exon 14 skipping events along with prominent ALK, BRAF, FGFR, NRG1, NTRK, RET, and ROS1 fusions and select point mutations in 14 key gene targets associated with lung cancer	AMP based	10 ng	Diagnosis
Lung Cancer-Targeted Gene Panel, Tumor	MAYO Clinic, Scottsdale, USA	DNA	EGFR, BRAF, KRAS, HRAS, NRAS, ALK, ERBB2, and MET	Amplicon based	NA	Diagnosis and management of lung cancer
Ion AmpliSeq™ Colon and Lung Research Panel v2	ThermoFisher Scientific, Waltham, USA	DNA	KRAS, EGFR, BRAF, PIK3CA, AKT1, ERBB2, PTEN, NRAS, STK11, MAP2K1, ALK, DDR2, CTNNB1, MET, TP53, SMAD4, FBXW7, FGFR3, NOTCH1, ERBB4, FGFR1, FGFR2	Amplicon based	10	Diagnosis and treatment selection
AmpliSeq for Illumina Colon and Lung Research Panel	Illumina, San Diego, USA	DNA	KRAS, EGFR, BRAF, PIK3CA, AKT1, ERBB2, PTEN, NRAS, STK11, MAP2K1, ALK, DDR2, CTNNB1, MET, TP53, SMAD4, FBXW7, FGFR3, NOTCH1, ERBB4, FGFR1, and FGFR2	Amplicon based	10	Diagnosis and treatment selection

**Table 3:** Studies describing the implication of NGS in lung cancer diagnosis.

Samples	Correlation with Other Techniques	NGS Method	Type of Lung Cancer	Specificity (%)	Sensitivity (%)	Ref.
31 tissues lung samples negative for mutations by FISH or PCR	8/31 presented actionable mutations	Broad, hybrid capture-based NGS	Adenocarcinoma	NA	NA	[70]
40 FFPE tissue with known fusion (test), 59 FFPE fusion-negative (validation)	Good concordance with FISH, PCR or Sanger	RNA seq gene fusion	NA	93–100	86–100	[71]
28 fusion positive FISH sample	16 were positive in NGS	RNA fusion and DNA seq	NSCLC	NA	NA	[72]
32 FFPE	Good concordance with FISH and qRT-PCR	RNA seq	NSCLC	100	100	[73]
50 FFPE (35 test positive for different fusion alterations, 15 negative) 109 FFPE (validation)	Good concordance with FISH	RNA fusion and DNA seq	NSCLC	100	100	[74]
31 FFPE positive for rearrangement by FISH	26 were positive in NGS and were confirmed by IHC	RNA fusion and DNA seq	NSCLC	NA	NA	[75]
51 tested with FISH, IHC and NGS	8 samples positive by NGS and IHC, only 4 by FISH	DNA seq	Adenocarcinoma	100	100	[76]
19 FFPE tested with IHC and NGS	Good concordance between NGS and IHC	DNA seq	Adenocarcinoma	NA	NA	[77]
63 tissue, urine and plasma	NGS testing of urine and plasma presented more EGFR mutated positive samples that tissue samples tested by RT-PCR	DNA seq	NSCLC	94 for urine 96–100 for plasma	80–93 for urine 87–100 for plasma	[78]
3 cases with multiple resected tumors	NGS revealed different molecular characteristics that the normal pathological diagnosis	DNA seq	Adenocarcinoma	NA	NA	[79]

**Table 4:** Clinical trials on lung cancer diagnosis using NGS technology.

Trial No	Condition	Scope of the Trial	Sample Type	Number of Patients	Results
NCT03558165	Lung adenocarcinoma stage IV	Diagnostic test: oncomine comprehensive assay	FFPE tissue	100	NA
NCT02420405	Non-squamous NSCLC stage IIIA-IV	Routine gene testing by NGS for diagnosis	Tissue	78	NA
NCT02297087	Incurable SCLC	Standard of care based on target(s) identified via GWAS for diagnosis and treatment	Blood and tissue	12	NA
NCT02281214	Bronchial adenocarcinoma with metastases, epidermoid cancer of the lungs	NGS testing for treatment selection and prognostic	Blood and tissue	165	NA
NCT03257735	NSCLC with brain metastasis	Consistency of gene mutation status between different types of samples using NGS	Cerebrospinal fluid, blood and tissue	50	NA
NCT04849481	NSCLC	Large-scale NGS analysis for novel treatment strategies and deciphering the mechanisms of drug resistance	Tissue	500	NA
NCT03244904	SCLC	NGS analysis for biomarkers for SCLC	Blood and tissue	80	NA
NCT02416726	Non-squamous NSCLC	NGS for gene profile comparison between different types of samples	Blood and tissue	35	NA
NCT04260295	Lung cancer and non-lung cancer patients	NGS for identification of microorganisms in lungs	Tissue	300	NA
NCT02705404	Multifocal lung cancer	NGS for differentiation of primary tumors from metastatic tumors	Blood, cytology and tissue	100	NA
NCT02705404	NSCLC	Targeted NGS for mutation profile concordance in different types of samples	Blood, fresh frozen and FFPE tissue	45	NA
NCT03833934	NSCLC	NGS testing for evaluation of ALK resistant mutations	Plasma	300	NA
NCT03220230	NSCLC	Concordance between NGS and IHC ALK status	Tissue and blood	4240	Accuracy 95.9% for 1450 participants, sensitivity 54.2% for 83 participants, specificity 98.4% for 1367 participants
NCT03658460	NSCLC	Gene testing using NGS with focus on immuno- oncology markers	Tissue	100	NA
NCT02273336	Lung cancer	NGS testing for treatment selection	Tissue, blood and cytology	40	NA
NCT02941003	Lung adenocarcinoma	NGS for early stage diagnosis	Tissue	540	NA
NCT04238130	NSCLC	NGS assessment of mutation profile in personalized analysis of cancer	Plasma	200	NA
NCT02169349	Stage IIIb and IV NSCLC	NGS evaluation cfDNA for diagnosis, treatment and disease progression	Plasma	100	NA
NCT02299622	NSCLC, head and neck cancer, esophageal cancer	NGS testing for evaluation of mutation profile	Tissue	200	NA
NCT02778854	NSCLC	Genetic detection of driver mutation using ddPCR and NGS for evaluation of the efficacy of liquid biopsy in diagnosis and prognosis	Tissue, plasma and other biological liquids	200	NA
NCT03486262	Lung carcinoma patients with/withoutidiopathic pulmonary fibrosis (IPH)	NGS testing for genetic alterations identification in lung cancer patients with IPH and without IPH	Tissue	100	NA
NCT02113852	NSCLC	NGS study for identification and characterization of genetic and transcriptomic alteration	Tissue and blood	250	NA
NCT03771404	Operable (stages I-IIIA) NSCLC Patients	NGS evaluation of the genetic landscape of each patient in order to determine heterogeneity in early stage NSCLC	Blood and tissue	50	NA
NCT04698681	Stage IV non-squamous NSCLC	NGS evaluation for tumor mutations identification in the KEAP1 or NRF2/NFE2L2 genes in order to determine potential eligibility for a biomarker selected clinical trial	Blood	200	NA
NCT04266483	NSCLC	Molecular typing of lung cancer in China	Blood and tissue	2500	NA
NCT04624373	Stage IV lung cancer	Molecular analysis to investigate the sensitivity of cytology supernatant DNA for genotyping	Supernatant, blood and tissue	50	NA
NCT02718651	NSCLC	New diagnostic test to detect ALK rearrangements using NGS	plasma	70	NA
NCT03576937	Non-squamous NSCLC	Comparison of blood-based mutational profile with tissue mutational profile for diagnosis	Blood and tissue	207	NA
NCT03248089	Non-squamous NSCLC	Investigation of the efficacy of cfDNA genotyping for diagnosis	Blood and tissue	186	NA
NCT03317080	I-IV lung cancer eligible for surgery.	Use of liquid biopsy for lung cancer detection	Blood	1500	NA
NCT04025515	Asian patients with NSCLC	Comprehensive molecular profiling of “actionable” alterations in lung cancer specimens in order to determine the prevalence of each genetic subtype in the local population.	Tissue	500	NA
NCT03706625	Immune-suppressed patients suffering from HIV-related NSCLC	Identify novel biomarkers such as tumor mutational profiling and immunomutanome in immunosuppressed patients	Tissue	170	NA
NCT03651986	Patients with benign and malignant pulmonary nodules	Development of a blood-based assay for early differentiation of benign and malignant pulmonary nodules	Blood	10,560	NA
NCT02906943	Several cancer including lung cancer	NGS evaluation of different types of cancer for biomarker identification	FFPE tissue	10,000	NA
NCT03609918	NSCLC	To build NSCLC gene mutation profile in China and find related correlation between gene mutation panel and clinical outcome	Fresh frozen tissues and FFPE tissues	513	NA
NCT03029065	Lung cancer patients with brain metastases	To determine whether cfDNA can be used for concomitant diagnosis to improve the treatment efficacy and prognosis of patients with brain (meningeal) metastasis	Tissue, plasma and cerebrospinal fluids	50	NA
NCT03971175	Lung cancer and relapse NSCLC	To evaluate accuracy of molecular genetic characterisation of NSCLC	Tissue, cytology and liquid biopsy	540	NA
NCT04692935	Lung adenocarcinoma from asian and Caucasian patients	Evaluation of the mutational profile by race	Tissue	450	NA

## Data Availability

Not applicable.

## Abbreviations

cfDNA: circulating free DNA; CGH: comparative genomic hybridization; ChIP Seq: chromatin immune-precipitation and DNA sequencing; CT: computer tomography; ddPCR: digital droplet polymerase chain reaction; DNA: deoxiribonucleic acid; FFPE: formalin fixed paraffin embedded; FISH: fluorescence in situ hybridization; GWAS: genome wide association studies; IHC: imunohisto chemistry; IPH: idiopathic pulmonary fibrosis; LC: lung cancer; LDCT: low dose computer tomography; miRNA: micro RNA; NCCN: National Comprehensive Cancer Network; NGS: next generation sequencing; NSCLC: non-small cell lung cancer; PCR: polymerase chain reaction; qRT-PCR: quantitative real time polymerase chain reaction; RNA: ribonucleic acid; SCLC: small cell lung cancer.
